# [Retracted] circANRIL reduces vascular endothelial injury, oxidative stress and inflammation in rats with coronary atherosclerosis

**DOI:** 10.3892/etm.2026.13158

**Published:** 2026-04-14

**Authors:** Peixia Shi, Hongling Ji, Huajuan Zhang, Jian Yang, Rui Guo, Jianwei Wang

Exp Ther Med 20:2245–2251, 2020; DOI: 10.3892/etm.2020.8956

Following the publication of this paper, it was drawn to the Editor's attention by a concerned reader that the immunohistochemical image shown in Fig. 1B on p. 2248 was strikingly similar to data appearing in different form that had already been published in an article in the journal *Medical Science Monitor* written by different authors at different research institutes. Moreover, upon performing an independent analysis of the data in this paper, it came to light that the ‘Negative group’ data panel shown in Fig. 2 on the same page subsequently appeared in an article in the journal *Bioengineered* that was also written by different authors at different research institutes, but which has since been retracted.

In view of the fact that the abovementioned data in Fig. 1 had already apparently been published previously, the Editor of *Experimental and Therapeutic Medicine* has decided that this paper should be retracted from the Journal. The authors were asked for an explanation to account for these concerns, but the Editorial Office did not receive a reply. The Editor apologizes to the readership for any inconvenience caused.

